# Systematic Review of Chemical Compounds with Immunomodulatory Action Isolated from African Medicinal Plants

**DOI:** 10.3390/molecules29092010

**Published:** 2024-04-26

**Authors:** Wendwaoga Arsène Nikiema, Moussa Ouédraogo, Windbedma Prisca Ouédraogo, Souleymane Fofana, Boris Honoré Amadou Ouédraogo, Talwendpanga Edwige Delma, Belem Amadé, Gambo Moustapha Abdoulaye, Aimé Serge Sawadogo, Raogo Ouédraogo, Rasmané Semde

**Affiliations:** 1 Laboratoire de Développement du Médicament, Ecole Doctorale Sciences et Santé, Université Joseph KI—ZERBO, 03 BP 7021 Ouagadougou 03, Burkina Faso; nikiemarsn@ujkz.bf (W.A.N.); windbedema.ouedraogo@ujkz.bf (W.P.O.); bigboris16.cforem@ujkz.bf (B.H.A.O.); edwige.delma@ujkz.bf (T.E.D.); hamade_belem@ujkz.bf (B.A.); moustapha_abdoulaye@ujkz.bf (G.M.A.); rasmane.semde@ujkz.bf (R.S.); 2 Centre d’Excellence Africain, Centre de Formation, de Recherche et d’Expertises en sciences du Médicament (CEA-CFOREM), Université Joseph KI—ZERBO, 03 BP 7021 Ouagadougou 03, Burkina Faso; fof.soul@ujkz.bf (S.F.); raogo.ouedraogo@ujkz.bf (R.O.); 3 Unité de Formation et de Recherche, Sciences de la Santé, Université Joseph KI—ZERBO, 03 BP 7021 Ouagadougou 03, Burkina Faso; serge.sawadogo@ujkz.bf; 4 Institut des Sciences de la Santé, Université NAZI Boni, 01 BP 1091 Bobo-Dioulasso 01, Burkina Faso

**Keywords:** medicinal plants, phytochemicals, immunomodulators, transduction mechanisms

## Abstract

A robust, well-functioning immune system is the cornerstone of good health. Various factors may influence the immune system’s effectiveness, potentially leading to immune system failure. This review aims to provide an overview of the structure and action of immunomodulators isolated from African medicinal plants. The research was conducted according to PRISMA guidelines. Full-text access research articles published in English up to December 2023, including plant characteristics, isolated phytochemicals, and immuno-modulatory activities, were screened. The chemical structures of the isolated compounds were generated using ChemDraw^®^ (version 12.0.1076), and convergent and distinctive signaling pathways were highlighted. These phytochemicals with demonstrated immunostimulatory activity include alkaloids (berberine, piperine, magnoflorine), polysaccharides (pectin, glucan, acemannan, CALB-4, GMP90-1), glycosides (syringin, cordifolioside, tinocordiside, aucubin), phenolic compounds (ferulic acid, vanillic acid, eupalitin), flavonoids (curcumin, centaurein, kaempferin, luteolin, guajaverin, etc.), terpenoids (oleanolic acid, ursolic acid, betulinic acid, boswellic acids, corosolic acid, nimbidin, andrographolides). These discussed compounds exert their effects through various mechanisms, targeting the modulation of MAPKs, PI3K-Akt, and NF-kB. These mechanisms can support the traditional use of medicinal plants to treat immune-related diseases. The outcomes of this overview are to provoke structural action optimization, to orient research on particular natural chemicals for managing inflammatory, infectious diseases and cancers, or to boost vaccine immunogenicity.

## 1. Introduction

The immune system comprises a complex network of cells and biological mediators that safeguard the body against harm from foreign invaders like microbes and malignant cell infiltration while preventing excessive immune activation. It is distinguished by innate and adaptive immunities, working synergistically to protect the body [1].

Several endogenous and exogenous factors may influence the immune system’s effectiveness, potentially leading to malfunction. In conditions such as infectious diseases, e.g., COVID-19, dengue fever, and autoimmune diseases, e.g., celiac disease, type 1 diabetes, Addison’s disease, Graves’ disease, and Rheumatoid polyarthritis, there is an inappropriate immune response [2,3,4]. For instance, the interaction of the dengue virus with immune cells triggers a cytokine storm (involving IL1β, IL6, and tumor necrosis factor α), exacerbating the disease [5]. Also, in autoimmune diseases, self-reactive T cells and the exaggerated production of antibodies against the body’s tissues result in persistent inflammation [6]. In both categories of diseases, controlling the immune response is crucial. 

Immunomodulation refers to any modification of the immune response and may involve the induction, expression, amplification, or inhibition of a part or phase of the immune response [7,8]. The concept of immunomodulation has gained significant attention, particularly with the resurgence of infectious diseases in recent years. Immunomodulators are categorized into immunostimulants, immunoadjuvants, and immunosuppressants. 

Immunostimulants are pharmacological agents capable of strengthening the body’s resistance to infection. In healthy individuals, they serve as preventive measures and potentiators by enhancing the immune response. They can be used in immunotherapy for individuals with compromised immune systems. Notably, immunostimulants show promise in cancer treatment [9]. Immunosuppressants are critical in preventing organ transplant rejection and managing autoimmune diseases and immune-related disorders linked to infections. Immunoadjuvants stimulate the immune system by enhancing the antigenicity of vaccines without exerting a specific antigenic effect. They serve three main functions: aiding in antigen-targeting immune cells, acting as depots for the gradual release of the antigen, and modulating and reinforcing the type of immune response induced. They can influence cellular and humoral response choices, Th1 and Th2, immune protection versus immune destruction, and regeneration [10,11]. These constitute a new and promising application for immunoadjuvants. 

Although synthetic immunomodulatory drugs offer many advantages, their undesirable side-effect profile and broad impact on the entire immune system are significant limitations to the extended use of these drugs, justifying the search for more effective and safer agents with targeted immunomodulatory activity. Using natural substances as immunoadjuvants during vaccine development to enhance immunogenicity is very promising [12]. Previous studies have shown that natural products with immunomodulatory activity have already been used to treat autoimmune diseases, inflammatory disorders, and cancer [13]. These substances constitute a valuable source of biologically active secondary metabolites, including alkaloids, polysaccharides, terpenoids, flavonoids, coumarins, glycosides, and proteins. This review aims to compile a comprehensive database of molecules from African medicinal plants capable of modulating immunity and improve our knowledge of the potential signaling pathways.

## 2. Results

### 2.1. Database Search Results

**Figure 1:**
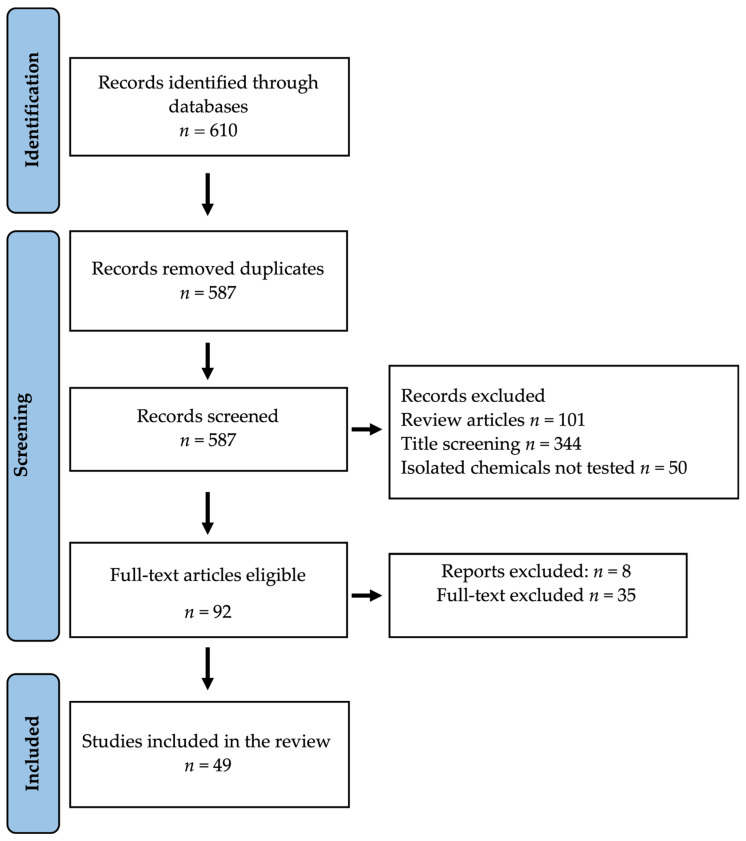
PRISMA flow diagram of the study selection.

In the course of our database search, a total of 610 articles were initially identified (Figure 1). Upon removing duplicate entries (*n* = 587), 495 studies were subsequently excluded due to their classification as review articles or because the studies focused solely on crude extracts without evaluating isolated bioactive substances. Consequently, we scrutinized 92 full-text articles for eligibility, ultimately excluding 43. Our final dataset comprised 49 studies investigating the immunomodulatory activity of African medicinal plants. 

The selected articles were published from 1994 to 2022. A total of 35 medicinal plant species belonging to 25 families were identified. The Liliaceae and Menispermaceae families were most represented with four and three plants species, respectively. 

From medicinal plants, 86 molecules were isolated, 80 molecules were evaluated for their immunomodulatory proprieties (Table 1), and 56 had their structures represented (Figures 3–7).

### 2.2. African Medicinal Plants Used for Immunomodulation

**Figure 2:**
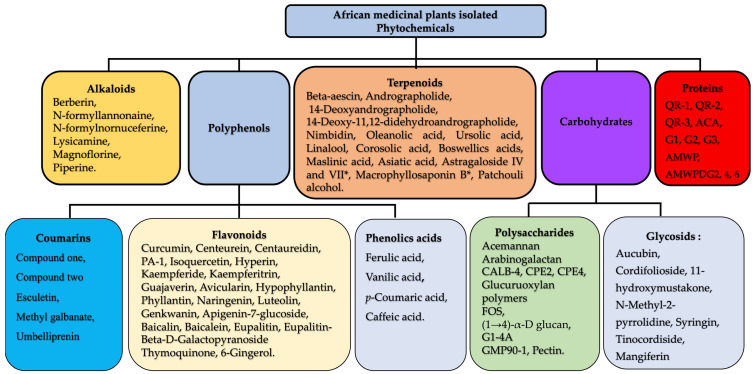
Plant- derived immunomodulators. The isolated phytochemicals with immunomodulatory activities include polyphenols (carbohydrates, alkaloids, and proteins). * NB: Astragalus VII and Macrophyllosaponin B were isolated from the Astragalus genius in particular *Astragalus trojanus Stev*. and *Astragalus oleifolius DC*, which are not distributed in Africa.

Numerous medicinal plants used in traditional medicine systems have attracted the attention of scientists worldwide (Table 1). As discussed below, these medicinal plants exhibit immunomodulatory activity due to various chemical groups (Figure 2) and others medicinal properties, including antioxidant, anti-inflammatory, analgesic, and anti-arthritic activity.

### 2.3. Chemistry of Plant-Derived Immunomodulators

#### 2.3.1. Alkaloids

**Figure 3:**
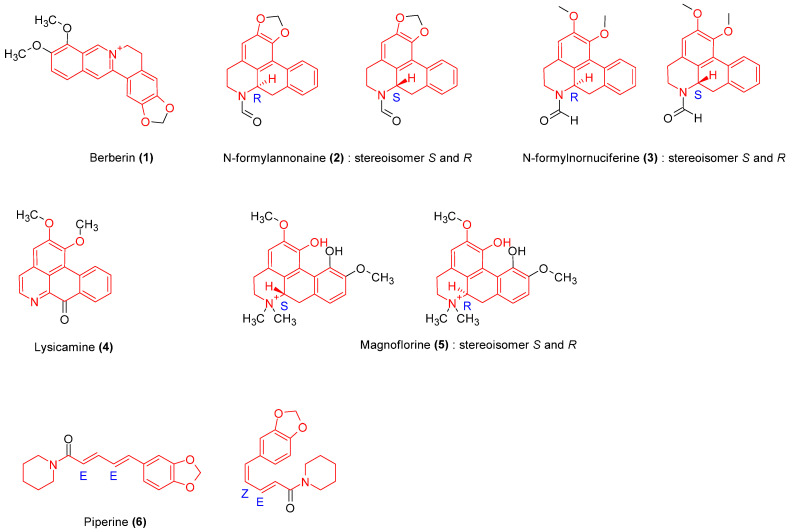
Structures of the identified alkaloids with immunomodulatory activities. The isolated alkaloids comprise a protoberberine isoquinoleic (**1**), aporphine isoquinoleine alkaloids (**2**–**5**), and a piperidine alkaloid (**6**). They have common scaffold highlighted in red. All have been tested and showed immunomodulatory activities.

Alkaloids isolated (Figure 3) from *C. pareira*, *T. crispa*, *T. cordifolia*, and *P. longum* exhibited immunomodulatory activities (Table 2).

Humoral immunity produces antigen-specific antibodies and is primarily driven by B cells. On the other hand, cell-mediated immunity does not depend on antibodies for its adaptive immune functions [75]. Mature T cells, macrophages, and the release of cytokines in response to an antigen primarily drive it. The alkaloid fraction of *C. pareira* tested on humoral and cell-mediated immunity by measuring the hemagglutination antibody titer and the delayed-type hypersensitivity (DTH) response demonstrated significant immunosuppressive effects at 25 to 100 mg/kg doses. It significantly (*p* < 0.01) reduced the humoral antibody titer and suppressed the DTH response (*p* < 0.01) at 75 mg/kg [76]. Berberine (compound **1**), an isoquinoline alkaloid isolated from *C. pareira*, displayed no effect on splenocyte proliferation. However, it downregulated the Th1/Th2 cytokines’ expression (TNF-α, IL-2, IL-4, IL-10) in a mouse primary splenocytes model assay at a range of concentrations (0.8, 1.6, and 3.3 μM, or 0.5 mL/well) [14]. 

Phagocytosis is an essential cell-defense mechanism against foreign, non-self organisms, and has been used as a critical non-specific immunological parameter to evaluate immune functions. Phagocytes also kill microbes via an oxygen-independent mechanism, although not as effectively as oxygen-dependent mechanisms. Macrophages are essential for the phagocytosis mechanism.

**Table 2:** Isolated alkaloids with immunomodulatory activities.

Isolated Molecules (n°)	Models	Pharmacodynamic Parameters ED_50_/IC_50_	Biological Effects	Cellular Effect	References
**1**	In vitro mouse primary splenocyte assay	6.6 µM	No significant effect on cell viability at 0.8, 1.6, and 3.3 μM	Downregulates splenocytes cytokines (IL2, 4, 10, TNFα) expression.	[14]
**2**	Mouse macrophage RAW 264.7 viability, chemotactic, phagocytic assay, ROS, NO, PGE2, and cytokine production, monocyte chemoattractant Protein-1 (MCP-1) production.	nd	Isolated compounds **2**, **3**, **4**, and **5** at concentrations above 25 μg/mL showed toxic effects on macrophages’ viability (<90%). Stimulation of cell migration. Increase in macrophage migration. Stimulation of cell migration, strong enhancement of macrophage phagocytic activity (81.01% compound **5**). Augmentation of ROS and NO generation.	Significantly stimulates PGE2 production, enhances the MCP-1 level. Significantly increases IL-1β, IL6, and TNFα production.	[17,18]
**3**		nd			
**4**		nd			
**5**		23.8 mM			
**6**	In vivo hematological assay, in vitro Dalton’s lymphoma ascites (DLA), Ehrlich ascites carcinoma (EAC) cells assay, L929 cells	nd	Increase in white cell count (138.9%), stimulation of stem cell proliferation, enhancement of the number of plaque-forming cells (71.4%) Cytotoxicity on DLA, EAC at 200 μg/mL, and L929 at 50 μg/mL.	Enhancement of the antibody production.	[21]

nd = non-determined.

In a murine RAW macrophages in vitro assay, the crude extract of *T. crispa* (concentrations of 25–200 μg/mL) and the isolated alkaloids (compounds **2**, **3**, **4**, and **5**) at various concentrations (1.56, 3.12, 6.25, 12.5, and 25 μg/mL) increased chemotactic activity and enhanced macrophage phagocytic activity. Furthermore, they significantly increased cytokine levels (TNFα, IL1β, and IL6) [17]. Cytokines like TNFα contribute to antitumoral effects.

*The treatment of* Balb/c mice with the alcoholic extract (10 mg/dose/animal) of fruits of *Piper longum* and piperine (compound **6**), a purified alkaloid, at 1.14 mg/dose/animal intraperitoneally for five consecutive days, yielded an increase in the white blood cell count by 142.8% and 138.9%, respectively. In addition, a cytotoxic effect was observed against L929 cancer cells at 100 µg/mL for the crude extract and 50 µg/mL for piperine [21].

#### 2.3.2. Polysaccharides 

Numerous studies have shown that plant polysaccharides can regulate the immune system in multiple ways and levels (Table 3). They not only activate immune cells, including T cells, B lymphocytes, macrophages, and dendritic cells, but they also activate and promote the production of cytokines (NO, TNFα, and IL6), thus showing regulatory effects on the immune system in various ways. 

Dendritic cells (DCs) act as initiators of the initial immune response and play an essential part in regulating the immune system [77]. DCs recognize, capture, process, and present antigens to naive T cells, which stimulate the activation and proliferation of naive T cells for adaptive immune responses. Assessing immunomodulatory activity on DCs has been performed. Polysaccharides isolated from *E. purpurea* and *Plantago asiatica* could upregulate the maturation of DCs [22,23,64]. They act on cell maturation markers by enhancing the expression of surface molecules, including CD80, CD86, and MHC class II.

The thymus is an organ of the immune system, and is the site of production and maturation of T lymphocytes. The spleen is the body’s largest secondary lymphoid organ and, as such, hosts a wide range of immunological functions in addition to its hematopoietic function [78]. In addition to B and T cells, a small amount of macrophages and other cells, such as dendritic cells, are included in splenocytes. The activity of plant extracts has been studied on spleen and thymus cells.

The immunomodulatory effects of plant polysaccharides on macrophages are mainly achieved through the generation of reactive oxygen species (ROS), the secretion of cytokines, cell proliferation, and the phagocytic activity of macrophages. A water-soluble pectic extract from *Allium cepa* exhibited the capacity to enhance NO production in murine macrophages and stimulate the proliferation of splenocytes and thymocytes. An optimal concentration for proliferation was observed at 50 μg/mL [57]. In vitro, fructo-oligosaccharides (FOS) provoked a significant increase in the mitogenic activity of murine splenocytes and thymocytes after 24 h incubation at 5 and 50 μg/mL concentrations. Macrophage activation is involved in the first phase of the immune response, and interestingly, onion FOS significantly induced macrophage phagocytosis and NO release [58]. A water-soluble glucan isolated from *M. oleifera* exhibited significant macrophage activation and phagocytic activity along with the induction of monocyte NO release at 0.1 μg/mL [74]. The effect of GMP90-1 polysaccharides isolated from *G. mangostana* on the viability of RAW 264.7 macrophage was studied using an MTT assay. These studies showed that GMP90-1 polysaccharides inhibited cell growth at 400 µg/mL, whereas 50–200 µg/mL concentrations had no inhibitory effect. Additionally, GMP90-1 polysaccharides increased macrophage phagocytosis and induced NO production and cytokine expression (TNF-α, IL-6, IL-1β) at concentrations of 50, 100, and 200 µg/mL [26].

**Table 3:** Isolated polysaccharides with immunomodulator activities.

Sources	Extraction Method	Molecular Weight (kDa)	Monosaccharide Composition	Active Substance	Biological Activity	References
*Allium cepa*	Hot water	1.8 × 10^2^	D-galactose: 6-O-Me-D-galactose: 3-O-acetyl-D-methyl galacturonate: D-methyl galacturonate1:1:1:1	Pectin	Enhancement of NO production in macrophage, stimulation of splenocyte and thymocyte proliferation.	[57]
	Hot ethanol			FOS: monosaccharide to hexasaccharide	Increase in splenocytes/thymocytes proliferation (~3-fold), macrophage phagocytic activity, NO production (~2.5-fold).	[58]
*Moringa oleifera*	Distilled water	70	Gluc.	(1→4)-α-D glucan	Increase in macrophage phagocytic activity, and in the number and percentage of globulin.	[74]
*Garcinia mangostana* L.	Water extraction	5.3	Ara., Gal., Rham.	GMP90-1 = arabinofurane	Enhancement of phagocytic activity (28.0%; 40.3% at 100 and 200 μg/mL, respectively), increase in NO secretion (2.2, 3.9, and 10.3 times at the concentrations of 50, 100, and 200, respectively), IL1β (38.42% at 200 μg/mL), IL6 (4.6, 5.1, and 8.5 times at 50, 100, and 200, respectively), TNFα (5.6, 41.7, and 200.1% at 50, 100, and 200 μg/mL, respectively).	[26]
*Aloe vera*	Distilled water	-	Man, Gluc, Gal. 62.9:13.1:0.6	Heteroglycan or acemannan	Increase in splenocyte proliferation (5.7 and 7.1% after 24 and 48 h, respectively). Increase in IL-1 and TNFα secretion in irradiated mice (2.34 and 1.32~fold, respectively).	[52]
*Echinaneae purpurea* L.	Water		Diploid, tetraploid	CPE2, CPE4	Stimulation of lymphocyte proliferation and cytokine secretion.	[24]
			Gal, Ara	Arabinogalactane		[79,80]
*Fructus aurantii*	Cold water, hot water	3.14 × 10^2^	Man, Rha, GlcUA, GalUA, Gal, Ara 16.3:4.0:2.9:3.4:21.7:41.7	Pectic polysaccharide CALB-4	Promotion of PBMC proliferation. Upregulation of NO production. Affects TNFα, IL1β, IL6, and IL8 secretion. Increases of proIL-1 expression.	[25]
*Siraitia grosvenorii*	Hot water		Gluc, Gal. Ara. Rham 5.8:0.77:0.38:0.12		Promotion of B and T lymphocyte proliferation. Increase in thymus index. Increase in IL-2 and decrease in IL-1.	[27]
*T. cordifolia*	Acetone extract			G1-4A	Upregulation of TNFα, IL1β, IL6, IL10, IL12, and IFNγ expression. Enhancement of NO level.	[20]
*Tamarindus indica*	Fresh water		Gal., Man., Gluc.		Increase in phagocytic activity. Inhibition of PHA-induced lymphocyte proliferation and leukocyte migration by 63–70.%	[72]
*Salvia officinalis* L.	Ethanol-water	10,000 < Mw > 50,000	Rham. Ara., Xyl., Man., Gluc., Gal., UA.	Arabinogalactans (A), Pectins (B), Glucurunoxylan polymers (D).	Polysaccharides-induced thymocyte proliferation.	[73]

Ara: arabinose; Gal: galactose; GalUA: galacturonic acid; Gluc: glucose; GlcUA: glucuronic acid; Man: manose; Rham: rhamnose; FOS: fructo-oligosaccharides; UA.: uronic acid.

Acemannan, a bioactive compound isolated from *A. vera*, displayed immunomodulatory activity. Studies in immunosuppressed mice indicated that acemannan treatment increased animal survivability and reduced mortality. It was established that acemannan upregulated cytokines’ (TNFα, IL1β, and IL6) production and improved peripheral lymphocyte counts, spleen cellularity, and the spleen index. Moreover, acemannan stimulated the macrophage nitric oxide release, surface molecule expression, and cell morphology in RAW 264.7 cells, a mouse macrophage cell line [52]. 

In vitro experiments demonstrated that tetraploid and diploid *E. purpura* enhanced the stimulation of mouse spleen lymphocytes by Concanavalin A. Tetraploid forms exhibited higher activity at lower concentrations and strongly promoted the release of IL-2 and IFNγ secretion [24].

Zampeng Shu et al. found that pectic polysaccharides (CALB-4) extracted from *F. aurantii* stimulated NO, TNF-α, IL-1β, IL-6, and IL-8 production depending on the concentration between 24 and 48 h. These polysaccharides also stimulated splenocyte proliferation and increased cyclophosphamide-induced carbon clearance [25]. Lymphocytes, which are essential contributors to the humoral immune response, were stimulated by polysaccharides extracted from *S. grosvenonii* [27]. These polysaccharides promoted splenocyte and thymocyte proliferation in an in vitro MTT model at 12.5 and 200 µg/mL concentrations. The effect on cytokine secretion was marked by a significant increase in IL-2 at 50 mg/kg and a notable decrease in IL-1β production at 400 mg/kg [27]. 

Polysaccharides isolated and purified from *T. indica* showed immunomodulatory activity by blocking mitotic activity induced by PHA on lymphocytes; they enhanced macrophages’ phagocytic activity, and inhibited leukocyte migration [72]. The immunomodulatory activity of water (A), ammonium oxalate (B), and potassium extractable (D) polysaccharide extracted from *S. officinalis* was evaluated via an in vitro co-mitogenic thymocyte test. Fraction A had an inhibitory effect at 300 μg/mL, and fractions B and D were at 1000 μg/mL. The inhibition was significant with fraction D. The optimum dose of this fraction was 100 μg/mL. Moreover, fraction D had a more marked SI_comit_/SI_mito_ rate (3–4) than fractions A and B (≈2) [73].

#### 2.3.3. Triterpenoids

**Figure 4:**
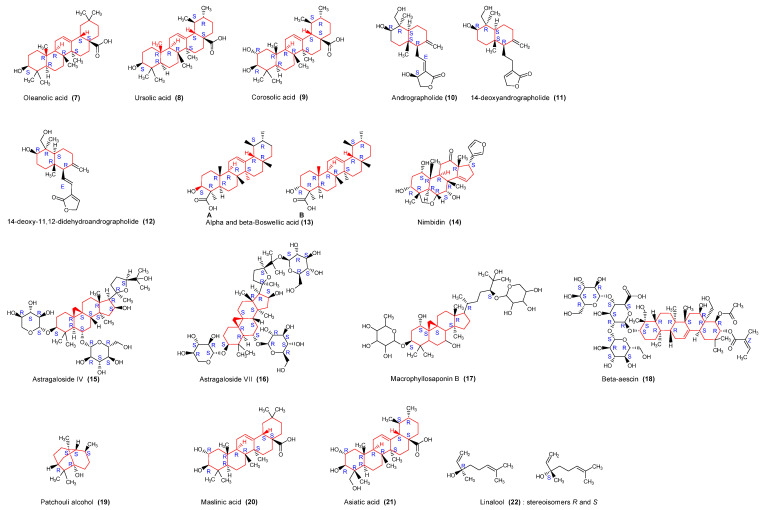
Structures of identified terpenoids with immunomodulatory activities. These terpenoids are distinguished in triterpenoid pentacyclic saponin (**7**, **8**, **9**, **13**, **20**, **21**), lactone sesquiterpenoid (**10**, **11**, **12**), tetracyclic saponin heterosid (**14**, **15**, **16**, **17**), pentacyclic saponin heterosid (**18**), diterpenoid (**19**) and Acyclic monoterpenoid (**22**). They have in common adjacent 2–5 rings, except for linalool (**22**). It is an advantage to have simple and complex structures like in linalool and beta-aescin (**18**), respectively, exhibiting immunomodulatory activities, which will lengthen the list of analogues.

Terpenoids, sometimes called isoprenoids, are a large and diverse class of naturally occurring organic chemicals that are similar to terpenes and are derived from assembled five-carbon isoprene units. Triterpenoids possess a rich chemistry and pharmacology with several pentacyclic motifs. They are used in inflammatory diseases and cancer therapeutics [81,82]. Numerous compounds (Figure 4) falling under the class of triterpenoids isolated from diverse medicinal plant species showed immunomodulatory properties.

Oleanolic acid (compound **7**) and ursolic acid (compound **8**): these pentacyclic terpenoids, extracted from various species of the Plantago genus, such as *P. major*, *Ocimum sanctum*, *Psydium guajava*, and *Phyllantus amarus*, have exhibited a range of pharmacological activities, including antioxidant and anti-inflammatory effects, functioning as immuno-inhibitors. Compounds **7** and **8** inhibited the peripheral blood proliferation of mononuclear cells (PMBCs) at 1.25 and 20 μg/mL, respectively. Ursolic acid displayed high activity at 40 μg/mL [63]. These compounds enhanced interferon-gamma (IFN-γ) secretion. In a model of human keratinocytes (HKLs), ursolic acid exhibited inhibitory effects on cell viability, while compounds **7** (15 μM) and **8** (30 μM) significantly increased respiratory burst levels. Both oleanolic acid (30 µM) and ursolic acid (30 µM) increased lysosomal enzyme activity, but ursolic acid (7.5 µM) inhibited lysosomal enzyme activity [46]. Corosolic acid (compound **9**) isolated from *P. guajava* reduced HKL cell viability and significantly enhanced the cell respiratory burst level after 24 h of incubation. It also increased lysozyme activity without affecting NO production. 

Andrographolides: in vivo and in vitro animal models were used to evaluate the immunomodulatory activity of these diterpenoids (compounds **10**–**12**) extracted from *A. paniculata*. Mice treated with different doses displayed a significant rise in the hemagglutination (HA) titer and in plaque-forming cells (PFC) in the spleen of sheep red blood cell (SRBC)-sensitized mice. Phagocytic activity assessed through carbon clearance exhibited a dose-dependent increase, and white blood cell counts were significantly increased [29]. 

The effect of compound **13** on the SRBC-induced delayed-type hypersensitivity (DTH) response indicated that oral administration inhibited the expression of the DTH response in mice. Splenocyte proliferation was inhibited at concentrations greater than 3.9 μg/mL, and the macrophage phagocytic activity function was enhanced [33].

Nimbidin (compound **14**), isolated from *A. indica*, possesses immunomodulatory activity by inhibiting macrophage cell migration, phagocytosis, and phorbol myristate acetate (PMA)-stimulated respiratory bursts. Nimbidin also exhibited inhibitory effects on IL-1β release and NO and PGE2 production [31].

*Astragalosides* (AST IV compound **15**; AST VII compound **16**): these cycloartane triterpenes with saponin-like structures are mainly extracted from the Astragalus genus species. AST VII and Macrophyllosaponin B (compound **17**) displayed low hemolytic activity at 500 µg/mL and increased splenocyte proliferation induced by Concanavalin A, lipopolysaccharide (LPS), and bovine serum albumin (BSA) in immunized mice. Immunoglobulin G1 and G2 antibody titers were increased by AST VII (120 µg) and Macrophyllosaponin B (90 µg), which stimulated IFNγ [83]. According to Nalbantsoy et al., Macrophyllosaponin B (156 µg/mL) exerts a suppressive effect on Th2 lymphocytes and a positive effect on Th1 lymphocytes by stimulating the release of specific cytokines (IL-2, IFNγ). It also inhibits the activity of inducible nitric oxide synthase (iNOS). In a murine model of a lymphoproliferation assay using MTT and a hemolysin spectrophotometry assay, AST IV increased T and B lymphocyte proliferation at 50–200 mg/kg. The activity of IL-1β at 1 nmol/L was increased. Additionally, TNF-α activity was inhibited with or without LPS stimulation [84].

β-aescin (compound **18**), isolated from the roots of *A. hippocastanum*, has demonstrated important antiviral and virucidal activity against the dengue and VSH viruses by targeting their envelope. The crude extract increased the secretion of pro-inflammatory cytokines (TNF-α and IL-6), while the extracted compound β-aescin from *A. hippocastanum* displayed a synergistic effect with glucocorticoids, enhancing anti-inflammatory activity. β-aescin, in an in vitro model of RAW264.7 cells, decreased the concentration of TNF-α, IL-1β, and NO in a concentration-dependent manner [28,85]. 

Compound **19**, a sesquiterpene tricyclic isolated from *P. cablin*, was studied for immunomodulatory activity in a mouse model. Oral administration significantly increased macrophage phagocytosis and boosted circulating immunoglobulin (IgM and IgG) while significantly decreasing the DTH response [34]. In the human peripheral blood mononuclear cells (PBMC) assay, compound **22** showed weak activity stimulating cell proliferation and a moderate stimulation of IFNγ secretion.

#### 2.3.4. Polyphenols 

The immune system plays a vital role in human well-being by increasing the immune response and providing protection. Polyphenols have well-demonstrated immunomodulatory effects, as they regulate the immune cells, macrophages, cytokines, and signaling pathways, and influence dendritic cells and lymphocytes (B and T), suppress T cell activation and natural killer cells, and suppress tumor-associated macrophages (Table 4).

**Figure 5:**
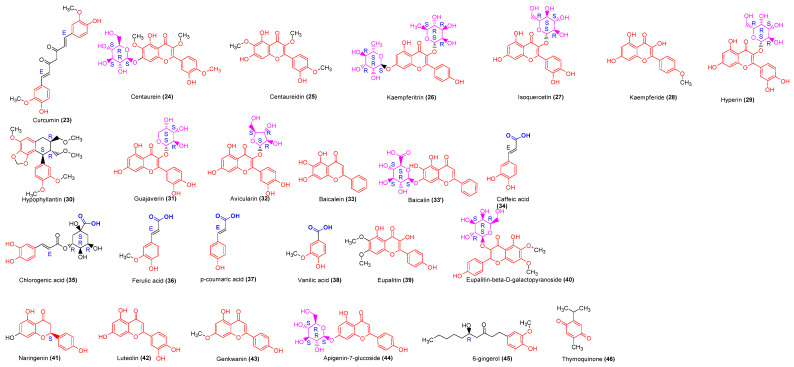
The structures of identified polyphenols acting as immunomodulators. The isolated polyphenols include flavonoids with the scaffold highlighted in red, polyphenolic acids with the acid group highlighted in blue, and ceto-phenolics. The thymoquinone is classed among polyphenols because in vivo metabolism gives phenolic compounds. The sugar group (highlighted in pink) is critical in the structure–activity relationship of flavonoids. Nevertheless, the active molecules have shown a similar way of modulating the immune system. They comprise phenol acid (**38**), cinnamic acid (**23**, **34**–**37**), lignan (**30**), flavonols (**25**, **28**, **39**), flavones (**33**, **42**, **43**), flavanone (**41**), heterosid flavonols (**24**, **26**, **27**, **29**, **31**, **32**, **40**), heterosid flavones (**33′**, **44**), and other phenolic compounds (**45**, **46**).

Polyphenols are a heterogeneous group of phenolic compounds with two major classes: flavonoids and phenolic acids (Figure 5). They show immunomodulatory activity.

**Table 4:** Polyphenols isolated from African medicinal plants acting as immunomodulators.

Isolated Molecules (n°)	Models	Pharmacodynamic Parameters		Biological Effects	Cellular Effect	References
		ED_50_	IC_50_			
**23**	Mouse macrophage, lymphocytes (PBMCs) proliferation assay, natural killer cytotoxicity assay	nd	nd	No effect on cell viability. Inhibition of lymphocyte proliferation. Enhancement of NK cytotoxicity	Inhibition of PHA-induced IL2 release, weak inhibition of TNFα production in PBMC	[38]
	Splenocytes assay	3.5 μg/mL		Inhibition of splenocyte proliferation	Inhibition IL-2 synthesis	[39]
**18**	PBMC cell proliferation assay		1.5 mg/mL	Suppression of lymphocyte proliferation		[36]
**24**, **25**	IFNγ promoter-driven luciferase reporter and T cells assay.	75 0.9 mg/mL		Modulation of IFNγ transcription		[35]
**27**, **28**, **29**	in vitro RAW 264.7 macrophage proliferation assay	nd	nd	Increase in macrophages’ proliferation (by 1.53-fold for compound **27** and 1.43-fold for compound **28**). No significant increase was observed for compound **29**.		[37]
**26**	Proliferation of murine splenocytes, macrophages, and human PBMCs. NO production, lysosomal enzyme activity, and neutral red uptake assay		nd	Increase in cell viability in the absence of LPS (macrophages 23%, splenocytes 17%, human PBMCs 24%). Increase in lysosome activity (57%) in a concentration-dependent manner. Lack of effect on neutral red uptake. Stimulation of NK cell activity (11%)	No effect on NO release.	[41]
**30**, **31**	HKLs	nd	nd	Modulation affects the viability of HKLs. No effect on cell viability Increase in lysozyme activity		[46]
**41**, **43**, **44**	In vitro mouse splenocyte proliferation assay, NK cell activity, cytotoxicity T lymphocyte activity, lysosomal enzyme activity	nd	nd	Induction of splenocyte proliferation in the presence or absence of mitogen. Enhancement of NK activity. Inhibition of lysosomal function in a dose-dependent manner	Reduction of NO production (from 53.37 μM to 22.33 μM for compound **44**; 20.66 μM for compound **43**; and 28.64 μM compound **41**)	[50]
**32**–**38**	Human PBMCs assay	nd nd	nd nd	Stimulation of PBMC proliferation	Stimulation of IFNγ secretion	[46]
**42**	Human PBMCs assay	nd	nd	Inhibition of cell proliferation	Inhibition of IL2 secretion. Inhibition of NO release	[71]
**39**, **40**	Human PBMCs assay, RAW cells assay	nd	nd	Inhibition of cell proliferation (n°39). Inhibition of lymphocyte proliferation. No effect on NK cytotoxicity		

nd = non-determined. The isolated polyphenol molecules with immunomodulatory activities reduced IL2 and NO secretion while increased IFNγ secretion.

Curcumin (compound **23**) has demonstrated an in vitro immunomodulatory effect. On human PBMCs, compound **23** isolated from *C. longa* exhibited no effect on cell viability but significantly inhibited PHA-stimulated lymphocyte proliferation and enhanced natural killer cytotoxicity. Curcumin inhibited PHA-stimulated IL-2 production and had a weak effect on TNFα release [38]. Another model evaluating curcumin (2.5 μg/mL) activity on splenocytes revealed an inhibition of mitogen-induced splenocyte proliferation and IL-2 synthesis [39]. Centaurein flavonoids: centaurein (compound **24**) and its aglycone centaureidin (compound **25**), isolated from n-butanol fractions of *B. pilosa*, were studied for immunomodulatory activity through a cell transfection model with plasmids. They demonstrated increased IFNγ and induced the nuclear factor of activated T-cells (NFAT) and NFκB activity [35]. Another flavonoid, PA-1 (IC_50_ = 1.25–2.5 µg/mL), exhibited inhibitory activity on lymphocyte proliferation in an in vitro model of murine lymphocyte stimulation [36].

The ethanolic extract of *B. diffusa* roots exhibited antiproliferative activity on various human and murine cell lines and human PBMCs. Two flavonoids (compounds **39** and **40**) were isolated from the ethanolic and chloroform extracts of *B. diffusa* roots. These compounds inhibited PBMC proliferation induced by PHA and the mixed lymphocyte reaction (MLR), natural killer cytotoxicity, and LPS-induced NO production. In RAW264.7 cells, compound **40** inhibited PHA-induced IL-2 release and LPS-induced TNFα [71,86].

Compound **26** at concentrations of 25 mM showed no toxic effect on murine splenocytes, macrophages, and human PBMCs. It induced cell proliferation, stimulated lysosomal activity, and increased the neutral red uptake in macrophages and the natural killer cell activity [41]. 

Flavonoids isolated from the ethanolic extract of *C. viridiflorus* leaves exhibited varying effects on RAW264.7 cells. Compounds **27** and **28** significantly increased cell proliferation, while compound **29** had no effect [87]. Certain flavonoids isolated from different extracts of *P. guajava* showed immunomodulatory activity in head-kidney leucocyte assays [46]. Hypophyllantin (compound **30**) and guajaverin (compound **31**) significantly affected cell viability, whereas avicularin (compound **32**) did not. Compound **30** significantly increased the production of RBA, whereas compound **31** did not. Compound **30** also significantly increased the NOS production and lysozyme activity in HKL after 24 h of contact.

Compounds **32** and **33**, isolated from *P. major*, exhibited significant (*p* < 0.05) human PBMC proliferation and IFNγ secretion stimulation. Compound **37** possesses intense activity stimulating human PBMC proliferation and IFNγ secretion (181 pg/mL). The stimulation index was 4.59. Compound **38** enhanced human PBMC proliferation at the range of 5 and 40 mg/mL concentrations. The activity of compound **36** was lesser than that of compound **37**; however, this compound possesses higher activity than compounds **34** and **36 [63]**. Compound **42** possesses inhibitory activity on human PBMC proliferation. The volatile oil extracted from *Z. officinale* (0.001–10 ng/mL) inhibited IL1α release in mice peritoneal macrophages. The DTH induced by DNFB was inhibited in a dose-dependent manner, and the inhibition rates were 31.6% (*p* < 0.01), 34.4% (*p* < 0.01), and 35.0 (*p* < 0.01), respectively. The thymus and spleen index decreased at 0.125, 0.25, and 0.5 g/kg bw doses. 6-gingerol (compound **45**) is a main pharmacologic substance isolated from *Z. officinale*. The combination of LPS and 6-gingerol had no significant cytotoxicity on RAW264.7 cells. Nitrite production was also significantly (*p* < 0.05) inhibited dose-dependently: 6-gingerol significantly suppressed iNOS proteins as well as mRNA levels, TNFα, and IL10 release. The molecule had a protective effect by preventing the calcium overload induced by H_2_O_2_. It blocked PKC-α translocation and suppressed LPS-induced cytoplasmic I-κBα phosphorylation [68,70]. The effect of thymoquinone (compound **46**), isolated from *N. sativa*, was evaluated on a rat *Wistar* cell proliferation model. A low concentration (1 μg/mL) did not significantly affect splenocyte viability and cell proliferation. At 5 μg/mL, a significant (*p* < 0.05) reduction of cell viability and proliferation was observed, whereas there was no effect on cytokines (IL4, IFNγ) production [67].

#### 2.3.5. Coumarins 

**Figure 6:**
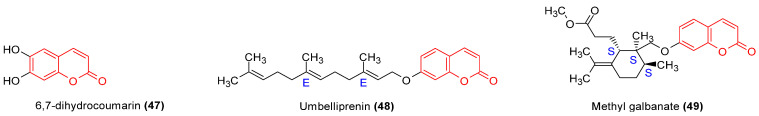
Structures of African medicinal isolated coumarins acting as immunomodulators. Sesquiterpenyl coumarins (**48**–**49**) with a long carbon chain show an identical mechanism of action while simple coumarin (**47**) has a different mechanism of action. The coumarin basic core is in red.

Coumarins isolated from various plant species (Figure 6) have demonstrated immunomodulatory effects (Table 5). Two coumarins isolated from *A. vera*, named compounds one and two were studied in an in vitro model. The compound one increased the macrophage phagocytic function in a concentration-dependent manner (50 to 200 µg/mL), with a maximum effect observed at 200 µg/mL, whereas compound two had no effect [51].

Esculetin (6,7-dihydrocoumarin, compound **47**), another isolated coumarin, from *A. vera* enhanced the mitogenic effect of splenocytes stimulated with LPS and concanavalin A. It induced lymphokine-activated killer (LAK) activity in lymphocytes [53]. 

Two coumarin terpenoids isolated from *F. szowitsiana*, compounds **48** and **49**, reduced PHA-induced splenocyte proliferation and preferentially induced IL-4 while suppressing IFNγ secretion [50].

**Table 5:** Isolated coumarins’ immunomodulator activities.

Isolated Molecules (n°)	Models	Pharmacodynamic Parameters		Biological Effects	Cellular Effect	References
		ED_50_	IC_50_			
**47**	Murine macrophages and lymphocytes assay	nd	nd	No effect on macrophage viability. Enhancement of endocytic activity induced by LPS on macrophages at concentrations of 80 and 120 mM. Increase in mutagenic-induced cell proliferation. Induction of LAK activity of splenic lymphocytes.	Enhances NO production and iNOS gene expression	[53]
**48**, **49**	Murine splenocytes assay	nd	nd	No effect on cell viability for tested concentrations (0.5–15 μM). Compound **48** at concentration >0.5 μM decreased splenocytes stimulation index. Compound **49** decreased cell proliferation at lowest dose. Suppression of PHA-induced cell proliferation.	Significantly augments IL4 secretion. Inhibits IFNγ production. Inhibits NO production by stimulated macrophages. Compound **48** increases PGE2 release; however, compound **49** inhibits it.	[50]

nd = non-determined.

#### 2.3.6. Other Molecules: Glycosides

**Figure 7:**
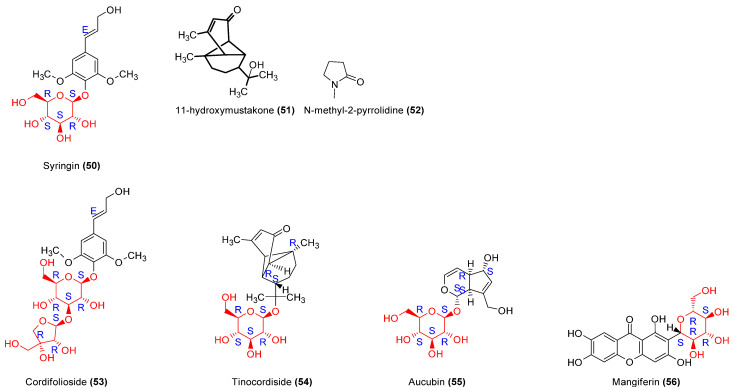
Structures of identified glycosides acting as immunomodulators. They comprise monosaccharides (**50**, **54**, **55**, **56**), disaccharides (**53**) and aglycans (**51**, **52**). The activity of glycosides is not linked only to the structure of the sugar unit (represented in red), but the aglycan part plays an important role. For example, the 11-hydroxymustakone molecule, without a sugar unit, has comparable activity to that of tinocordiside and cordifolioside.

Syringin (compound **50**), a phenolic glucoside isolated from *T. crispa* and *T. cordifolia (*Figure 7), was investigated in an in vitro study using RAW 264.7 mouse macrophage cultures. It significantly reduced macrophage phagocytic activity and cell chemotaxis. The impact on cytokine production included reducing TNFα, IL1β, and IL6 production [17,18].

Compounds **51**, **52**, **53**, and **54** (Figure 7 and Table 6) increased cell phagocytic activity and NO production in cells [18]. Compound **55** tested on PBMC showed significant stimulation of cell proliferation and enhanced IFNγ secretion [63].

Mangiferin (compound **56**), an isolated natural xanthone glucoside, demonstrated immunomodulatory activity. The 100 mg/kg dose significantly increased IgG and IgM levels, whereas IgA levels decreased in in vivo mice model [66].

**Table 6:** Isolated glycosides’ immunomodulator activity.

Isolated Molecules (n°)	Models	Pharmacodynamic Parameters		Biological Effects	Cellular Effect	References
		ED_50_	IC_50_			
**50**	Murine RAW 264.7 cell viability assay Chemotaxis assay Phagocytosis assay NO, ROS, PGE2 production Monocyte chemoattractant Protein-1 production Cytokine production	nd	nd	Toxicity effect above 25 μg/mL. Reduction of cell chemotactic and phagocytosis activities. Diminution of MCP-1 production (IC_50_ = 48.3)	Reduction of NO production. Inhibition of PGE2 production (IC50 = 12.08 μM). Decrease in IL1β, IL6, and TNFα production.	[17]
**51**, **52**, **53**, **54**	PMN cells viability assay Phagocytosis assay ROS, NO production assay	nd	nd	Increase in phagocytosis activity	Dose-dependent increase in NO and superoxide production	[18]
**55**	Human mononuclear cells assay Lymphocytes transformations test	nd	nd	Stimulation of PBMC proliferation	Enhancement of IFN-γ production	[63]

nd = non-determined.

#### 2.3.7. Proteins

The analysis of raw garlic extract revealed the presence of several proteins within the 10–75 kD range (Table 7). The mitogenic activity on peripheral blood lymphocytes (PBL) demonstrated a significant increase in cell proliferation at 10 mg/mL concentrations. Notably, protein QR-2 exhibited the highest mitogenic activity. Additionally, the modulatory effect on splenocytes and thymocytes displayed a stimulatory effect on cell proliferation, while QR-1 and -2 showed agglutination in rabbit erythrocytes [54]. 

The thymus is the primary immune organ that produces functional T cells. The effect of onion agglutinin (ACA) on thymocyte proliferation showed an 4- and 3.5-fold increase in cell proliferation at 0.01 μg/well and 0.1 μg/well, respectively, at 24 h. On the other hand, ACA showed a weak increase in LPS-induced B-lymphocyte proliferation (1.3-fold). It significantly elevated the expression of IL-2 and IFN-γ. The macrophages are the first line of defense of the body against infections. At 0.1 μg/well, ACA induces a significant increase (6–8-fold) in NO production by RAW264.7 at 24 h. The release of cytokines (TNFα and IL-12) was significantly stimulated [59]. 

Proteins isolated from *A. membranaceus* displayed immunomodulatory activities. These proteins significantly affected the proliferation of splenocytes, murine peritoneal macrophages, and bone marrow-derived dendritic cells (BMDCs) at 10–90 µg/mL, except at 10 µg/mL. The optimal activity was observed at a concentration of 50 μg/mL. These proteins promoted the phagocytosis effect of murine peritoneal macrophages, with the compound AMWPDG2 displaying the highest activity. Furthermore, these proteins significantly promoted the secretion of various cytokines and chemokines, including TNFα, IL-6, IL12p40, IL-1β, IL-1α, nitric oxide, hydrogen peroxide, and CXCL1 and CXCL3 secretion [61].

**Table 7:** Isolated proteins with immunomodulator activity.

Sources	Extraction Method	Isolated Proteins	Molecular Weight (kDa)	Biological Effects	References
*Allium sativum*		QR-1, QR-2, QR3 (7:28:1)	13	Mitogenic activity on human PBMC, murine splenocytes and thymocytes. QR-1 and QR-2 showed hemagglutination and mannose-binding activities.	[54]
*Allium cepa*	Dialysis-D-mannose chromatography	ACA: *Allium cepa* Agglutinin	12	ACA at 0.1 μg/well and 0.01 μg/well enhance thymocyte proliferation by ~4- and 3.5-fold, respectively, with a marginal effect on B cells proliferation (~1.3-fold at 0.01 μg/well), significantly increased cytokine production (TNFα, IL12), and IFN-γ and IL2 expression. ACA induced an ~8-fold increase in NO production by rat peritoneal cells at 12 and 24 h. ACA (0.01–10 μg/well) significantly enhanced IL12 (~3-fold) and TNFα (~2–3-fold) release. The phagocytosis activity is enhanced by 2-fold by ACA (0.1; 1; 10 μg).	[59]
*Tinospora cordifolia*	Chromatography	G1, G2, G3	10–80	The proteins at a concentration range of 1–10 μg/mL showed mitogenic activity (3-fold) in murine splenocytes at 1–10 μg/mL and ~5–7-fold in thymocytes. They induced NO release by macrophages and enhanced macrophage phagocytosis activity.	[19]
*Astragalus membranaceus*,	Alkali extraction	AMWP (16 aa) AMWPDG2 (16 aa), AMWPDG4 (15 aa), AMWPDG6 (15 aa)	- 406.115 268.795 342.281	All proteins contain seven essential amino acids: Thr, Val., Met., Ile., Leu., Phe., and Lys. Proteins at 50 μg/mL significantly promoted in murine peritoneal macrophage phagocytosis activity, secretion of immunomodulatory factors like NO (AMWPDG2 > AMWPDG4 = AMWPDG6) and H_2_O_2_ (AMWPDG2 > AMWPDG6 > AMWPDG4) and inflammatory cytokines (TNFa and IL6)	[61]

aa: amino acid; Thr.: threonine; Val.: valine; Ile: Isoleucine; Leu: leucine; Phe.: phenylalanine; Lys.: lysine; Met.: methionine.

### 2.4. Mechanism of Action of Plant-Derived Immunomodulators 

Numerous studies have elucidated the molecular mechanism underlying the immunomodulatory effects of phytochemicals. These compounds activate macrophages and other cells, such as dendritic and lymphocyte cells, through Toll-like Receptors (TLR) [88]. Proinflammatory cytokines and other immune system mediators are closely associated with the induction of transcription factors, including NF-kB, the nuclear factor of activated T lymphocytes, signal transduction, and transcription activator (STAT). 

Cell signaling is initiated by receptor stimulation, with most receptors belonging to the TLR family. TLRs are pivotal receptors for many natural substances, including lipopolysaccharides (LPS), natural polysaccharides, alkaloids, and terpenoids. The activation of TLRs triggers the recruitment of MyD88 and subsequently activates specific intracellular pathways. All TLR signaling pathways ultimately lead to the activation of the transcription factor NF-kappa B, which regulates the expression of numerous inflammatory cytokine genes. Three main cell-signaling pathways, including phosphoinositide (PI3K-Akt), Mitogen-Activated Protein Kinases (MAPKs), and nuclear factor kappa B (NF-kB), can activate and transcribe NF-kB.

The MAPK pathway consists of a three-tier kinase cascade in which MAP3K activates MAP2K, which activates MAPKs. MAPK signaling pathways include the activation of the extracellular-related kinase (ERK1/2), p38 isoforms (p38), and c-Jun NH2-terminal kinase (JNK1/2). Once activated, MAPKs can be phosphorylated and translocated into the nucleus, leading to the expression of related genes and cellular responses, such as the secretion of signaling molecules (NO and ROS) and cytokines (IL-1β, IL6, TNFα). 

In resting cells, NF-κB in the cytoplasm tightly associates with the inhibitory protein IκB, forming the IκB kinase (IKK) complex. Activation of the NF-κB signaling pathway provokes the disintegration of the IKK complex, leading to the phosphorylation and degradation of IκB-α. Consequently, NF-κB is released, translocated into the nucleus, and bound to DNA, initiating the transcription of proinflammatory-related genes. 

The isolated phytochemicals target many proteins in these signaling ways (Table 8). Oleanolic and ursolic acid inhibit TLR4. Oleanolic acid blocks TLR3 activation and inhibits mRNA expression while suppressing the activation of IKKα/β proteins [89]. Magnoflorine and G1-4A activate MAPKs while being inhibited by flavonoids, such as immunomodulator curcumin.

**Figure 8:**
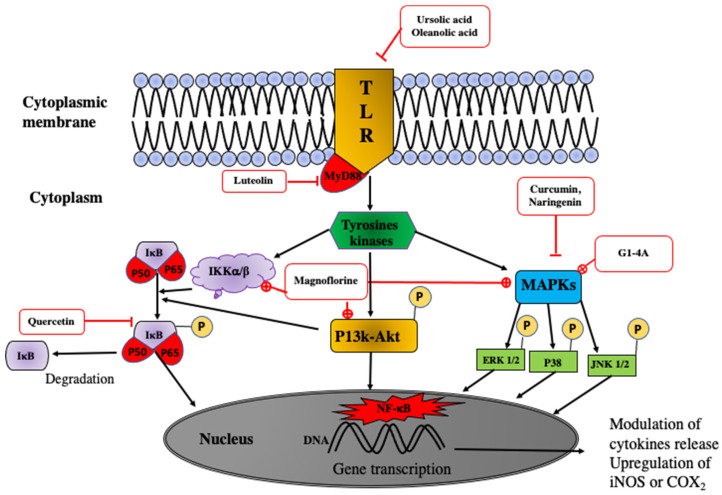
Immunomodulatory signal-transduction pathways of some molecules. TLR: toll-like receptor; MyD88: myeloid differentiation primary response gene 88; MAPKs: mitogen-activated protein kinases; P13k-Akt: phosphatidylinositol-3-kinase-kinase B protein; Ik-Bk: inhibitory kappa B kinase; IKK: I-kB kinase; p38, p50, p65: proteins 38, 50, 65; P: phosphate; NF-kB: nuclear factor kappa B; ERK: extracellular signal-regulated kinase; JNK: c-Jun N-terminal kinase; 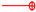: activation or stimulation by phytochemical; 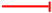: inhibition or reduction by phytochemical; →: transduction way or activation.

Ursolic acid and betulinic acid inhibit the degradation of Iκ-Bα, the phosphorylation of IkBα and p64 protein, the activation of Iκ-Bα kinase, and the translocation of p65 [90]. Astragaloside IV activates the phosphorylation of the p65, p38, ERK, and JNK proteins [91]. Magnoflorine, an alkaloid extracted from *T. crispa*, activates the phosphorylation of p65 and increases the phosphorylation and degradation of IκB. It also increases the phosphorylation of the JNK, ERK, and p38 proteins [92]. Curcumin inhibits LPS-induced NF-kB activation by suppressing the MAPK pathway [93]. The possible molecular mechanisms of isolated bioactive-induced immunomodulation are shown in Figure 8 and Table 8.

**Table 8:** Convergent mechanism of action of isolated compounds of African medicinal plants with immunomodulator activities.

Compounds N°	Phytochemical Group	Cellular Model	Receptor	Transduction Pathway	Mechanism of Action	Cellular Actions	References
**5**	Alkaloids	Macrophages (U937)	TLR4	MAPKs, PI3K-Akt	Augmentation of Akt phosphorylation, induction of JNK, ERK, and p38 phosphorylation	Enhancement of upregulation of TNFα, IL1β, PGE2, COX-2	[92]
**G1-4A**	Polysaccharides	Macrophages	TLR4/MyD88	MAPKs	Activation of JNK, ERK, and p38 phosphorylation	Upregulation of the expression of TNFα, IL6, IL12, IL10	[20]
**8**	Triterpenoids	Macrophages	TLR4	TLR4- MyD88	Blocking TLR4/MyD88	Decrease in TNF-α, IL-1β et IL-6 release	[94]
**7**	Triterpenoids	THP1 cells	TLR3	MAPKs	Inhibition of IκB phosphorylation and NF-κB translocation		[89]
**15**	Terpenoids saponins	Macrophages		MAPKs/NFκB	Increase in the phosphorylation of p65, p38, JNK, and ERK, and a decrease in their protein expression	Increase in IL1β, IL6, TNFα, and inducible nitric oxide synthase	[91]
**23**	Flavonoids	Dendritic cells		MAPKs/NFκB	Suppression of MAPKs and p65 activation	Reduction of inducible NO synthase and IL-12	[93]
**27**	Flavonoids	Macrophages	TLR4	MAPKs	Suppression of phosphorylation of proteins p50/p65	Increase in TNFα, IL1β, iNOS	[95]
**55**	Glycosides	3T3-L1 adipocytes		NF-κB	Suppression of ERK phosphorylation and IκBα degradation	Inhibiting TNFα production	[96]
**56**	Glycosides	Mouse primary hepatocytes		MAPKs	Inhibiting the activation of c-JNK and ERK ½		[97]

The proinflammatory mediators such TNFα, IL1β, IL6, IL10, COX-2, PGE2, and NO release are inversely modulated by inhibitors (**7**, **8**, **23**, **55**, **56**) and activators (**5**, **15**, **27**, **G1-4A**).

## 3. Discussion and Perspectives

This systematic review reported the immunomodulatory properties of 86 isolated phytochemicals or groups from 35 medicinal plant species belonging to 25 different families of African flora. Fifty-seven molecules had their structures identified and tested for immunomodulatory properties. 

The review covered data from accessible full-text publications and did not take into account grey literature or others’ protected data. Nevertheless, based on the obtained data, an analysis of the chemical-based immunomodulatory properties of isolated molecules in various immune cell models, including macrophages, splenocytes, thymocytes, T and B lymphocytes, dendritic cells, and human PBMCs, was carried out. 

The chemical structures of the isolated immunomodulators are organized according to phytochemical groups. These molecules offer a wide range of therapeutic options, such as treating immune-related inflammation, cancer, and oxidant stress-related diseases. 

### 3.1. Alkaloids

Among the identified alkaloids, berberine, piperine, and magnoflorine showed relevant prospects. 

Studies have shown that berberine, an iso-quinoline isolated from various medicinal plants, has a variety of pharmacological actions, including antiarthritic, antibacterial, and anticancer effects [98,99]. Berberine is active on several cancer cell lines and has remarkable antiviral activity on several viruses, justified by its immunomodulatory mechanism. 

Berberine has been reported to attenuate the radio-resistance of colon cancer cells by repressing P-gp expression [100] and to sensitize breast cancer cells to different chemotherapeutic drugs [101]. Berberine also attenuates ovarian cancer cell resistance to cisplatin (DDP) by targeting the miRNA-21 to regulate the post-transcriptional expression of the tumor suppressor programmed cell death 4 [102]. Pretreatment with berberine promoted the antitumor effects of DDP on laryngeal cancer cells [103]. Pandey et al. showed the potential actions of berberine in attenuating resistance to 5-fluorouracil in gastric cancer cells [104].

The isoquinoline alkaloids have multi-target potential in multifactorial chronic diseases, including immune, metabolic, and neurological disorders, and present a high perspective in therapy [105]. 

The alkaloids also had antiviral activities on both RNA and DNA viruses. Their broad spectrum of activity is of interest for identifying new applications, like for berberine, an isoquinoline with clinical promise. Berberine effectively disrupts the replication process of various DNA and RNA viruses, including the human immunodeficiency virus and the new severe acute respiratory syndrome linked to coronavirus-2 (SARCOV-2) [106,107,108]. 

Berberine targets antiviral activity by inhibiting RTase [109] with an IC_50_ = 0.13 mM on HIV-1 NL4.3, activating protein kinase signaling pathways in several viruses (see the figure describing the mechanism of action: the kinase cascade) or the ERK and JNK signaling cascades to prevent the generation of virions. By activating the MAPK pathway, berberine reduces the virion titer during CHIKV infection [110] and suppresses RSV replication [111].

Piperine, another alkaloid isolated from *P. longa*, showed antitumoral, anti-inflammatory activities correlated with its immunomodulatory activity. It inhibited the translocation of NF-κB subunits like p50, p65, and c-Rel, as well as CREB, ATF-2, and c-Fos [112]. In an in vitro LPS-induced osteoarthritis model, piperine treatment showed anti-inflammatory activity by downregulating miR-127 and MyD88 expression. Piperine triggers apoptosis in ovarian cancer cells by increasing the JNK and p38 MAPK phosphorylation [113].

Based on these results, isolated alkaloids could be candidates for therapeutic agents for diseases-based NF-κB signaling pathways’ activation.

### 3.2. Polysaccharides and Proteins

Peritoneal macrophages possess important immunological functions, such as immune defense, surveillance, regulation, and antigen presentation [114]. Phagocytosis is the first step in the macrophage response to invading microorganisms, and the activation of phagocytosis elevates the innate immune response. Activated peritoneal macrophages induce NO production, and various immunostimulatory factors, such as IL-6 and TNF-*α*, play important roles in the phagocytosis, antigen presentation, and inflammatory regulation of macrophages [115]. It is important to measure the activity of immunostimulatory factors to highlight the immune-based mechanism of protection or pathophysiology genesis of immune-related diseases to guide medication. Numerous plant-isolated molecules act on macrophages, enhancing phagocytic activity and stimulating NO or TNFα release. The spleen is the largest immune organ reflecting the systemic immune status in the human body, and it plays an important role in anti-infection and anticancer activities [116]. The proliferation of spleen lymphocytes is key to activating cellular and humoral immunomodulatory responses. Some proteins (QR-1, QR-2, QR-3, AMWP, AMWPDG2, AMWPDG4, and AMWPDG6) and polysaccharides (Pectin, FOS, Glucan, CPE2, CPE4, and CALB-4) stimulate splenocytes’ and thymocytes’ proliferation. Polysaccharides help boost the immune system while also possessing anti-tumor properties. 

### 3.3. Terpenoids

Among the terpenoids studied, oleanolic acid, ursolic acid, and betulinic acid have divergent effects depending on the concentration. Oleanolic acid stimulates T lymphocyte proliferation at 0.5 μg/mL, while ursolic and betulinic acid have an inhibitory effect with IC_50_ of 3.01 and 50 μg/mL, respectively [117]. Depending on their concentration, molecules belonging to the same phytochemical family can have opposite effects. 

The chemical structure plays a significant role in the biological effect observed. Studying the structure–activity relationship will help elucidate the mechanism. Ursolic and oleanolic acids correlate with different biological activities, such as anti-inflammatory, anticancer, and antidiabetic [118]. Different mechanisms, including immunomodulation, could justify these proprieties. 

Immunomodulators act by stimulating or suppressing the immune response. Therefore, they can be used in several therapeutic areas. 

In vaccine development, new and improved adjuvants are needed to mitigate adverse effects and increase immunogenicity. AST VII and Mac B isolated from Astragalus have shown an immunostimulant effect by increasing IgG and IgG1 antibody titers with a smaller hemolytic effect. They also act by activating T and B lymphocytes during immunization with BSA [83]. The glucuronoxylan-D polymer isolated from *S*. *officinalis*, which has a significant comitogenic effect, has potential adjuvant properties [73].

Immunomodulators offer exciting prospects in the treatment of cancer. Those that strengthen the body’s ability to identify and eliminate cancer cells are revolutionizing treatment and can also help to strengthen the immune system. Immunomodulators have great potential in the treatment of autoimmune diseases. They, therefore, help to restore immunological balance. 

They also offer excellent prospects for treating infectious diseases and combating bacterial resistance. B-aescin isolated from the seeds of *A. hippocastanum* has virucidal and antiviral activity in addition to its immunomodulatory effect [119]. Combining these effects would provide comprehensive treatment while strengthening the immune system.

### 3.4. Polyphenols 

The negative impacts of synthetic drugs and the quest for natural alternatives for therapy have led to an increased demand for the multi-target action of phenols for enhancing immunity [120].

Combining quercetin with piperine, flavonoid, and no-flavonoid compounds presented an effective and potent anti-inflammatory strategy for treating acute colitis in mice. This anti-inflammatory effect was mediated by impaired DC immune responses [121]. Several flavonoids also display antitumor effects directly or indirectly. Luteolin enhanced the sensitivity of lapatinib in human breast cancer cells, and the combination of baicalein and cisplatin increased the apoptosis of gastric cancer and A549 lung adenocarcinoma cells in vitro [122,123].

The immunomodulatory effect is one of the most important mechanisms for the anti-tumor effect of flavonoids. They enhanced the cytotoxicity of NK and CTL cells to tumor cells via the upregulation of their activating receptor [124,125]. 

Flavonoids inhibit the production of various pro-inflammatory cytokines (IL6 and IL1β). Undeniably, the inflammatory tumor microenvironment is essential in the progression of malignant [126]. Baicalein (**33**) and baicalin (**33′**) are directly cytotoxic to some tumors. In addition to direct cytotoxicity, these two flavonoids stimulate the T cell-mediated immune response against tumors by reducing PD-L1 expression in cancer cells [127]. Kaempferol, curcumin, and quercetin inhibit the proliferation of numerous cell line [128].

NF-κB is essential in human cancer initiation, development, metastasis, and treatment resistance [129,130,131,132]. Many human cancers exhibit constitutive NF-κB activity due to the inflammatory microenvironment and various oncogenic mutations. NF-κB activity is associated with tumor cell proliferation [132]. In addition to suppressing apoptosis and promoting angiogenesis, it also induces an epithelial–mesenchymal transition, facilitating distant metastasis formation. NF-κB activation can also remodel the local metabolism and stimulate the immune system to promote tumor growth. The suppression of NF-κB in myeloid or tumor cells generally results in tumor regression, making the NF-κB pathway a promising therapeutic target. Howeve r, due to its vital role in various biological activities, selective targeting of components of the NF-κB pathway must be achieved for therapeutic purposes. 

Since NF-κB plays an essential role in both tumor cells and the tumor microenvironment, targeting NF-κB as an anticancer therapy has been explored extensively over the last few decades. Hundreds of natural and synthetic compounds have been reported to inhibit NF-κB. However, their clinical application has shown little efficacy, except for certain types of lymphoma and leukemia [132]. 

Flavonoids such as curcumin, naringenin, and quercetin inhibit NF-κB through various signaling pathways, which could justify their anticancer activity. 

### 3.5. Glycosides

The immunomodulatory properties of mangiferin have been demonstrated in several studies. It inhibits NF-KB by reducing the translocation of the p65 protein subunit. It also inhibits activation of the AGE-RAGE/mitogen-activated protein kinase (MAPK), the c-Jun N-terminal kinase (JNK), and the p38 pathways. The expression of extracellular regulated kinase 1/2 (ERK1/2) in the myocardium is also increased. Mangiferin could have beneficial effects in diabetic cardiomyopathy [133,134]. Mangiferin has antioxidant and anti-apoptotic properties via the MAPK/NF-κB/mitochondria-dependent pathways. It has an immunoprotective effect during cancer therapy [65,135]. Obesity is closely associated with a state of chronic inflammation, characterized by the abnormal production of cytokines and the activation of inflammatory signaling pathways in adipose tissue. The signaling pathways involve MAPKs. 

Aucubin (**55**) suppressed the activation of extracellular signal-regulated kinase (ERK), the degradation of inhibitory kappa Ba (IκBα), and the subsequent activation of nuclear factor kappa B (NF-κB) [96]. Aucubin could improve obesity-induced atherosclerosis by attenuating TNF-α-induced inflammatory responses.

## 4. Methods 

### 4.1. Search Strategy

The research was conducted according to PRISMA guidelines to identify studies about African medicinal plants used for their immunomodulatory properties. This exploration involved using the scholarly search engine Google Scholar and a comprehensive screening of prominent international databases, including PubMed, ScienceDirect, African Journal Online, and Embase. Our search queries incorporated specific keywords such as “immunomodulator” OR “immunity” AND “medicinal plant” OR “herbal plant’’ AND “phytochemicals”. 

We exclusively considered scientific research articles published in English until December 2023, ensuring that they were accessible without restrictions. Article titles and abstracts were screened according to the research objectives, which focused on the immunomodulatory effects of African medicinal plant chemicals. This examination encompassed instances involving either full or partial isolation, coupled with the subsequent determination of the chemical structure of active compounds. Articles deemed relevant following independent evaluation were included.

### 4.2. Data Extraction

Eligible records were extracted into Microsoft Excel, 2013 by NWA and double-checked by OM, OWP, and RO. We systematically gathered data from each publication, including scientific names, botanical families, used parts of plants, extraction solvents, phytochemical groups, isolated compounds, and experimental models for objectifying immunomodulatory activities. These data were meticulously documented using a standardized Excel sheet form. 

The risk of bias and the quality of each article were assessed by two independent reviewers.

Following this, we analyzed proposed mechanisms underpinning immunomodulatory activities to elucidate convergent and distinctive signaling pathways associated with the active compounds. The chemical structures of the isolated compounds were generated using ChemDraw^®^ (version 12.0.1076). The specific scaffold (alkaloids, terpenoid, phenol, polyphenol, coumarin, or glycoside) was drawn in red, the sugar group and acid group were respectively drawn in pink and blue. In purpose to demonstrate stereochemistry, R and S chirality centers configuration were determined using the Cahn Ingold Prelog priority rules, and the E and Z absolute configurations for double bounds were indicated.

This systematic review protocol is registered on inplasy.com at number INPLASY202410116 (Accessed on 29 January 2024).

## 5. Conclusions

The present review is a contribution to highlighting African medicinal plants with isolated immunomodulatory molecules. The isolated chemicals, such oleanolic acid, ursolic acid, boswellic acid, betulinic acid, astragalosides, magnoflorine, luteolin, curcumin, andrographolides, centeurein, centaureidin, quercetin, guaverin, corosolic acid, naringenin, pectin, acemannan, nimbidin, syringin, esculetin, umbeliprenin, methyl galbanate, hypophyllantin, and baicalein, possess significant immunomodulatory properties. These molecules belong to various phytochemical groups: alkaloids, polyphenols, terpenoids, carbohydrates, glycosides, and proteins.

The chemical structures’ stereoisomers’ demonstration of the isolated compounds allows an analytical approach to the structure–activity relationship, as their main immunomodulatory transduction pathways are proposed. The pharmacological properties of the chemicals can be improved by chemical group optimizing to yield analogs with improved pharmacokinetic and pharmacodynamic properties.

The chemical actions comprise modulating the expression or phosphorylation status of various accessory proteins associated with the TLRs, STAT3, NF-κB, MAPKs, and PI3K/Akt pathways. The therapeutic perspectives of such immunomodulators are infectious, cancer, and chronic inflammatory diseases’ care, and the development of immunoadjuvants for vaccines.

The review data can also contribute to the registration of immunomodulatory plant-based traditional medicines, to orient researchers towards the screening of new immunomodulatory chemicals from the species or genera of other medicinal plants, or to allow the comparison with immunomodulatory medicinal plants and phytochemicals of other continents.

## Figures and Tables

**Table 1:** African medicinal plants-based isolated immunomodulators.

Species and Families of Plants	Parts of Plant Used	Solvent	Chemical Groups	Isolated Molecules	Other Biological Activity	Reference
*Cissampelos pareira* L., Menispermaceae	Roots	Methanol	Alkaloids	Berberine (**1**), Tetrandrine.	Antioxidant, antibacterial	[14,15]
*Tinospora crispa*, Menispermaceae	Stem	Ethanol	Alkaloids, glycosides, terpenoids	N-formyllannonaine (**2**), N-formylnornuceferine (**3**), Lysicamine (**4**) Magnoflorine (**5**) Syringin (**50**) 1-Octacosanol.	Anti-inflammatory, antioxidant	[16,17]
*Tinospora cordifolia* (*Wild) Hook. F.* & *Thomson*, Menispermaceae	Stem	Methanol, n-hexane, chloroform, ethyl acetate and n-butanol	Alkaloids, glycosides, proteins	11-hydroxymustakone (**51**), N-methyl-2-pyrrolidone (**52**), N-formylannonaine (**2**) Cordifolioside (**53**), Tinocordiside (**54**) Syringin (**50**)		[18,19,20]
*Piper longum Linn*. Piperaceae	Fruits	Methanol	Alkaloids	Piperine (**6**)	Anti-inflammatory, anti-infectious, antitumor, analgesic	[21]
*Echinacea purpura*, Echinaceae	Whole plant, Root	Methanol, ethanol, aqueous	Polysaccharides, flavonoids	Polysaccharides, alkyl amides, Arabinogalactans, Caffeic acid(**34**)	Antioxidant, anti-inflammatory	[22,23,24]
*Fructus aurantii*, Rutaceae	Fruit		Polysaccharides	Pectic polysaccharide: CALB-4	Anti-carcinogenic, antimicrobial	[25]
*Garcinia mangostana* L., Guttiferae	Bark	Methanol	Polysaccharides	Arabinofuran (GMP90-1)	Antioxidant, anti-inflammatory, antimicrobial	[26]
*Siraitia grosvenorii*, Cucurbitaceae	Whole plant	Aqueous	Polysaccharides	Polysaccharides	Antioxidant, anti-inflammatory	[27]
*Aesculus hippocastanum*, Hippocastanaceae	Seed	Alcoholic	Saponins triterpenoides	β-aescin (**18**)	Antiviral	[28]
*Andrographis paniculata*, Acanthaceae	Whole plant	Methanol-water	Terpenoids	Andrographolide (**10**) 14-deoxyandrographolide (**11**); 14-deoxy-11,12-didehydroandrographolide (**12**),	Anticancer, Anti-inflammatory	[29,30]
Azadirachta *indica*, Meliaceae	Oil		Terpenoids	Nimbidin (**14**)	Anti-inflammatory, anti-arthritic	[31]
Ocimum *sanctum* Lamiaceae	Whole plant	Alcoholic, aqueous	Terpenoids, essential oils, phenols, flavonoids	Eugenol, Carvacrol, Oleanolic acid (**7**), Ursolic acid (**8**),	Anti-inflammatory, antiallergic	[32]
*Boswellia serrata Roxb*. Burseraceae	Oleogum resin		Terpenoids	Boswellic acids (**13**)	Anti-inflammatory	[33]
*Pogostemon cablin* Benth. Lamiaceae	Aerial parts	Ethanol aqueous	Terpenoids	PA: Patchouli alcoholic (**19**)	Antioxidant, Antimicrobial	[34]
*Biden Pilosa*, Asteraceae	Whole plant	n-butanol	Flavonoids	Polyacetylene 2-O-β-D-glucosyltrideca-11^E^-en-3,5,7,9-tetrayn-1,2-diol (PA-1), Centaurein (**24**), Centaureidin (**25**)	Anti-inflammatory, antihyperglycemic	[35,36]
*Callistenom viridiflorus*, Myrtaceae	Leaves	Ethanol	Phenols, flavonoids	Apigenin 4′-O-β-d-glucopyranosyl- (1″’ → 4″)-O-β-d-glucopyranoside, Kaempferide (**28**), Isoquercetin (**27**), Hyperin (**29**)	Anti-inflammatory, analgesic, antibacterial, antifungal.	[37]
*Curcuma longa*, Zingiberaceae	Rhizome		Flavonoids	Curcumin (**23**)	Anti-inflammatory, antimutagenic	[38,39]
*Justicia spicigera* *Schltdl*. Acanthaceae	Leaves	Ethanol	Flavonoids	Kaempferitrin (**26**)	Antioxidant, antitumor	[40,41]
*Phyllantus amarus*, Euphorbiaceae	Leaves	Ethanol, fractions: ethyl acetate, dichloromethane	Flavonoids, lignan	Corosolic acid (**9**), Oleanolic acid (**7**), Phyllanthin, Hypophyllanthin (**30**)	Anti-inflammatory, antiviral, antimutagenic.	[42,43,44]
*Psidium guajava*, *M*yrtaceae	Leaves	Ethanol	Flavonoids, glycosides, phenolic compounds, terpenoids	Ellagic acid, Hyperin (**29**), Isoquercitin (**27**), Guajaverin (**31**), Avicularin (**32**), Asiatic acid (**21**), Maslinic acid (**20**), Corosolic acid (**9**), Oleanolic acid (**7**), Ursolic acid (**8**)	Antiallergic, antitumoral, anti-inflammatory, analgesic, antimicrobial	[45,46]
*Teucrium ramosissimum Desf.*, Lamiaceae	Aerials parts	Chloroform	Flavonoids	Apigenin-7-glucoside (**44**), Genkwanin (**43**) Naringenin (**41**)	Antioxidant, anti-inflammatory	[47,48]
*Ferula szowitsiana*, Apiaceae	Roots	Methanol	Coumarins terpenoids	Methyl galbanate (**49**), Umbelliprenin (**48**)	Anti-inflammatory, antioxidant	[49,50]
*Aloe vera*, Liliaceae	Whole roots	Chloroform	Coumarins, flavonoids, phenolics, carbohydrates, lignans	Esculetin (6,7-dihydrocoumarin) (**47**) Acemannann	Anti-inflammatory, antioxidant	[51,52,53]
*Allium sativum*, Alliaceae	Bulbs	PBS	Proteins	Proteins (QR-1, QR-2, QR-3), Fructans, proteins (QA-1, QA-2, QA-3)	Anti-inflammatory, antioxidant, antimicrobial, antitumor	[54,55,56]
*Allium cepa*, *Alliaceae*	Bulbs	Ethanol	Proteins, polysaccharides, lectins	Pectin, FOS (fructo-oligosaccharides), Agglutinin	Antimicrobial	[57,58,59]
*Astragalus membranaceus*, Fabaceae	Waste	Alkali solvent	Proteins, saponins, alkaloids, polysaccharides, glucosides	Proteins: AMWPDG2, AMWPDG4, AMWPDG6, Astragaloside IV (**15**), Astragaloside VII (**16**), Macrophyllosaponin B (**17**)	Immunoadjuvants	[60,61,62]
Plantago sp. (*P. major*, *P. asiatica*) Plantiginaceae.	Leaves	Aqueous	Flavonoids, phenols, terpenoids, iridoids,	Aucubin (**55**), Chlorogenic acid (**35**), Ferulic acid (**36**), p-Coumaric acid (**37**), Vanillic acid (**38**), Luteolin (**42**), Ursolic acid (**8**), Oleanolic acid (**7**), Baicalein (**33**), Baicalin (**33′**).	Anticancer, antimicrobial, anti-inflammatory, antioxidant	[63,64]
*Mangifera indica* L. Anacardiaceae	Leaves	-	Xanthone glucoside	Mangiferin (**56**)	Antioxidant, antitumoral	[65,66]
*Nigella sativa* L. Ranunculaceae	Seeds	Ethanolic	Volatile oil	Thymoquinone (**46**)	Anti-inflammatory, antioxidant.	[67]
*Zingiber officinale * Zingiberaceae	Dried ginger	Distilled water	Volatile oil, polyphenols	6-Gingerol (**45**)	Antibacterial, anti-inflammatory, antitumoral	[68,69,70]
*Boerhavia diffusa* Nyctaginaceae	Leaves	Hexane, chloroform, ethanol	Flavonoids	Eupalitin (BdI) (**39**), Eupalitin-3-O-β-D-galactopyranoside (BdII) (**40**)	Anti-inflammatory	[71]
*Tamarindus indica* Leguminoseae	Seeds	Water	Polysaccharides	Polysaccharides	Antitumoral	[72]
*Salvia officinalis* L. Lamiaceae	Arial parts	Methanol-chloroform	Polysaccharides, proteins	Arabinogalactans (A), Pectins (B), Glucurunoxylan polymers (D).	Anti-inflammatory	[73]
*Moringa oleifera*, Moringaceae	Mature pods	Aqueous	Polysaccharides	(1→4)-α-D glucan	Anti-inflammatory	[74]

The chemical compounds or groups (*n* = 86) were isolated from 35 medicinal plants species belonging to 25 different families. The numbers in parentheses are the appearance order numbers in the text of the 57 isolated chemicals whose structures were drawn (Figures 3–7).

## Data Availability

Any supplementary information can be obtained on request addressed to the corresponding author.
